# Parental Awareness and Attitude about Childhood Immunization in Riyadh, Saudi Arabia: A Cross-Sectional Study

**DOI:** 10.3390/ijerph18168455

**Published:** 2021-08-10

**Authors:** Shuaa Z. Alshammari, Isamme AlFayyad, Youssef Altannir, Mohamad Al-Tannir

**Affiliations:** 1 Research Center, King Fahad Medical City, Riyadh 11525, Saudi Arabia; szalshammari@kfmc.med.sa (S.Z.A.); ialfayyad@kfmc.med.sa (I.A.); 2 College of Medicine, Alfaisal University, P.O. Box 50927, Riyadh 11533, Saudi Arabia; yaltannir@alfaisal.edu

**Keywords:** parent, awareness, attitude, childhood, immunization, vaccination, Saudi Arabia

## Abstract

Parental beliefs about vaccination are one of the main factors in reaching high vaccination rates. This cross-sectional study aims to assess the awareness and attitudes regarding routine childhood immunization among Saudi parents in Riyadh, Saudi Arabia. This survey, with a pretested 18-item questionnaire, was conducted on parents having at least one child from Riyadh, Saudi Arabia, between 1 May 2019 and 1 November 2019. The validated questionnaire consisted of three sections; participants’ demographics, awareness, and attitude regarding the immunization of their children. In total, 1200 parents participated in the study, 883 (73.3%) of the parents scored a good knowledge of childhood immunization, and 93% knew that routine vaccination protects children from infectious diseases and their complications. Around 10% stated that immunization can cause autism. Only parents in age groups 30–39 and 40–49 were 1.76 (*p* < 0.05) times and 1.92 (*p* < 0.05) times, respectively, more likely to exhibit good knowledge. About 522 (43.6) of the parents attained a positive attitude toward immunization. Adherence to the immunization schedule was confirmed important by 93%, while 91% presumed that immunization keeps their children healthy. Additionally, immunization was perceived as important by 94% of parents and only 8% agreed that immunization is prohibited by religion. Females were 1.45 (*p* < 0.05) times more likely to exhibit positive attitudes than males. Parents have good knowledge and a positive attitude towards child immunization. However, parental education should be focused on the fact that religion supports immunization, and more awareness should be focused on the lack of correlation between autism and vaccination.

## 1. Introduction

Childhood vaccination is considered as one of the most effective public health strategies of the 20th century, to control and prevent infectious diseases [1,2]. A vaccine is composed of weakened or dead bacteria, or viruses, that are administered to individuals to stimulate their immune system to protect them against bacterial, viral, or fungal infections [3]. The main goal of childhood vaccination strategies is to achieve disease reduction by reaching high levels of immunity, and to prevent the spread of serious childhood diseases via proper immunization coverage [4]. Therefore, vaccinated children are protected against more than a dozen infectious diseases such as tetanus, diphtheria, measles, polio, and pertussis [5].

The national immunization program was launched in Saudi Arabia in 1979. Diphtheria, tetanus, and pertussis (DTP) were the first vaccines introduced and it was expanded to include additional vaccines [6]. Remarkably, vaccination is considered as a prerequisite for school entry at the age of six years in Saudi Arabia [6]. However, according to the “WHO vaccine-preventable diseases: monitoring system” in 2019, the vaccination coverage rates for those in Saudi Arabia was 52% for BCG compared to 98% in 2018, and the coverage rates for DTP and measles, mumps, and rubella vaccine (MMR) were 98% and 96%, respectively. Despite a high immunization rate, the number of reported cases in 2019 was 1035 for measles, 187 for mumps, 326 for pertussis, and 62 for rubella [7].

Nevertheless, the high vaccination rate may not infer Saudi public confidence in vaccines, possibly for two reasons. First, the compulsory prerequisite of a comprehensive vaccination for children’s admission to school, is almost certainly impelling parents’ decisions to vaccinate their children. Second, parents still have uncertainties and misconceptions related to childhood vaccines [6]. Moreover, other reasons have been reported in the literature that undermine the vaccination rate, which include medical, socioeconomic, religious, or philosophical reasons [8,9,10].

Parents are the primary and essential health decision makers for their children and the sole determinants, and most important advocates, in realizing increasing compliance rates and the achievement of complete vaccination programs. Hence, their knowledge and attitude regarding immunization have a significant impact on the vaccination status of their children [11,12]. Therefore, a periodic and continuous assessment of public knowledge and attitudes towards childhood immunization is needed to address the main areas needing improvement, and to tailor targeted interventions to change perceptions, which ultimately ensures the success of vaccination programs [13,14]. Thus, this cross-sectional study aims to assess the awareness and attitude regarding routine childhood immunization among Saudi parents in Riyadh, Saudi Arabia.

## 2. Materials and Methods

### 2.1. Study Design, Settings, and Subjects

This cross-sectional study was conducted from 1 May 2019 to 1 December 2019 in 10 selected elementary schools across the five regions of Riyadh province, Saudi Arabia (East, West, North, South, and Central). Parents were selected using convenience sampling, regardless of their socio-demographic characteristics. The respondents were briefed with an information sheet explaining the aim of the study, voluntary participation, and assuring their identities would be kept anonymous and confidential. Following the parents’ consent, a self-administered questionnaire was used to collect parents’ information about their knowledge and attitudes toward childhood vaccination.

### 2.2. Data Collection

The study questionnaire was developed by the research team based on the frequently asked questions and answers published by the Saudi Ministry of Health concerning vaccination [15]. The questionnaire was initially designed in English and underwent linguistic validation using a forward-backward translation technique by expert translators, and was reconciled by an expert panel. The questionnaire was administered to the participants in the Arabic language. A face validity testing was also conducted by expert researchers and medical physicians to assess the comprehensiveness of the designed questions, and the clarity of wording. Following the face validity testing, a pilot study was performed on 30 parents to compute the reliability of the questionnaire. A Cronbach alpha test showed a high score of >0.7 for the knowledge and attitude scales.

The questionnaire comprised of two parts. Part 1 sought the socio-demographic characteristics of the parents, and part 2 collected data on parents’ knowledge and attitude towards childhood vaccination. The parents’ knowledge was explored with a structured questionnaire of 9 questions, on a three-point Likert scale, ranging from “I do not know”, “No”, and “Yes”. For the purpose of analysis, parents who answered “No” and “I do not know” were considered an indicator of lacking the knowledge, and were combined and coded with a “0” score, and “Yes” answers were coded with a score of “1”.

A five-point Likert scale (“Strongly Agree”, “Agree”, “Not Sure”, “Disagree”, and “Strongly Disagree”) was used to assess parents’ attitudes (8 questions) towards childhood immunization. For the purpose of analysis, the “Strongly Agree” and “Agree” were combined and coded with a score of “3”, “Disagree” and “Strongly Disagree” were also combined and coded with a score of “1”, and “Not Sure” answers were coded with a score of “2”.

The total score of knowledge ranged from 1 to 9, and the total score for attitude ranged from 1 to 8. The threshold median score for the questionnaire was considered as 7 for knowledge and 5 for attitude. A score of ≥7 was considered good knowledge and ≥5 was considered a positive attitude, respectively.

The sample size was estimated following the assumption that 85% of the parents would have a positive attitude [16], a confidence interval of 95%, and a margin error of ±5%. The sample size was calculated to be 1200 participants, which was adjusted up to 1440 to ensure attaining the required number of responses, considering a 20% drop out rate.

## 3. Statistical Analysis

Data were analyzed using SPSS version 21.0 (IBM Corporation, Armonk, NY, USA). Descriptive statistics (mean, standard deviation, and percentages) were used to describe the quantitative and categorical variables. Association between dependent variables (knowledge and attitudes) and independent variables (parents’ demographics) was tested using the chi-square test. Binominal regression analyses were performed to identify the predictors of good knowledge and positive attitude towards vaccination. *p*-values of < 0.05 were considered statistically significant.

## 4. Ethical Consideration

Ethical approval for the study protocol was obtained from the Institutional Review Board at King Fahad Medical City, with the log number: 19–249, and written informed consent was obtained from the participants before enrollment in the study.

## 5. Results

Overall 1200 parents were enrolled, of whom 585 (48.8%) were females. The majority of the respondents were distributed in the age groups 30–39 and 40–49; 481 (41.1%) and 310 (25.8%), respectively. Since the study was conducted in Riyadh province, which is considered an urban city and is the capital city of Saudi Arabia, the number of parents with university qualifications was on the higher side. Table 1 shows the parents’ characteristics.

## 6. Parental Knowledge on Childhood Immunization

The majority of parents, 1116 (93%), were aware of the role of routine vaccination in protecting children from infectious diseases and its complications, and 1041 (86.8%) parents knew that the first dose of vaccination is given at birth. Eight hundred and seventy-six parents knew that most diseases against which children are vaccinated occur during the first years of life. Around 70% of parents knew that the administration of two or more doses of the same vaccine is important for child immunity. Nearly half of the parents knew that providing more than one vaccine simultaneously had no negative impacts on child immunity. Table 2 shows detailed parents’ knowledge on childhood immunization.

## 7. Parents’ Attitude towards Childhood Immunization

The vast majority of the parents, 1123 (93.7%), either strongly agreed or agreed that child immunization is important, and the majority considered immunization to be helpful and safe, 1075 (89.7%), and 1002 (83.6%), respectively. A minority of parents, 96 (8.0%), believe that it is forbidden in Islam. Moreover, 353 (29.5%) of parents were not certain if an immunized child becomes infected with the disease against which he/she was vaccinated against or not. Table 3 shows detailed parents’ attitudes toward childhood immunization.

Table 4 shows that 883 (73.3%) of the parents scored a good knowledge on immunization. The results revealed that parents’ age and the number of children were significantly associated with their knowledge of immunization. Logistic regression was conducted to ascertain the effects of age and the number of children on the likelihood that parents have good knowledge. Only parents in age groups 30–39 and 40–49 were 1.76 (95% CI: 1.04–2.98) times and 1.92 (95% CI: 1.12–3.32) times, respectively, more likely to exhibit good knowledge. Furthermore, 522 (43.6%) parents attained a positive attitude towards immunization. Only the parents’ gender was significantly associated with their attitudes towards immunization. Logistic regression was conducted to ascertain the effects of gender on the likelihood that parents have positive attitudes. Females were 1.45 (95% CI: 1.15–1.82) times more likely to exhibit positive attitudes than males.

## 8. Discussion

Parental knowledge of childhood immunization is crucial for increasing the vaccination coverage rate and parents’ compliance with the vaccination schedule [17]. Based on our study findings, the majority of the respondents showed good knowledge of childhood vaccination in protecting children against potentially infectious diseases and their complications. These findings were consistent with those findings reported from low, middle, and high-income countries [16,18,19,20,21,22]. This finding is very important as poor or inadequate parental knowledge of the health benefits of vaccines, in protecting children against infectious diseases, is associated with low parental confidence and vaccination coverage rates [23,24]. Moreover, this finding is a key quality metric of the positive impact of the immunization program in Saudi Arabia on public health.

Most of the vaccines in the immunization schedule need two or more doses for the development of sufficient and persistent antibody response [6]. Parents enrolled in this study knew the importance of multi-dose vaccines to boost children’s immunity. For producing adequate and permanent antibody response, the Centre for Disease Control and Prevention (CDC) asserts that most vaccines require several doses [25]. This result aligns with views reported in previous studies from Malaysia and Saudi Arabia [17,26]. Moreover, nearly one-third of parents considered that vaccination is contraindicated during common colds, ear infections, and diarrheas. The guide for contraindications to childhood vaccination indicated that misconceptions about contraindications may delay the vaccination and expose the children to avoidable health risks [27].

Although several systematic reviews emphasized that there was no scientific link between measles, mumps, and rubella (MMR) vaccine and autism [28,29,30], the hypothesized association between the measles, mumps, and rubella (MMR) vaccine, and child autism remains challenging to vaccine uptake worldwide [28]. A significant proportion of our study respondents believed that childhood vaccines can cause autism or they have no information about this debate, though it was adduced by them that multi-doses are beneficial in boosting the immune response of children. However, this dilemma is common globally, even in developed countries. Half the studies included in the systematic review about parents’ beliefs around childhood vaccines in the United States believed that vaccines can cause autism [31]. The influence of media coverage of the link between the MMR vaccine and autism received significant public attention, and thus vaccination rates fell in many countries [32,33,34]. Therefore, robust monitoring and evaluation of the internet and social media, along with formal education campaigns in Saudi Arabia, are warranted to prevent disseminating misleading information about vaccines’ side effects from non-medical sources.

Recent recommendations in the United States have stated that immunizing all children from the age of six-months up to 19-years with an emphasis on children under the age of five-years or with chronic illnesses with influenza vaccines [35]. Nearly 45% of the parents knew that the immunization of children against seasonal influenza is beneficial. Parents would be interested in immunizing their children if educated about the essential role of children in communicating the infection in families and in public, as well as the economic burden and health effects of contracting influenza [6].

Although the bivariate analysis showed that parents’ age and the number of children had a significant association with good knowledge, the regression model revealed that parents aged 49 and younger had better knowledge. These findings are very significant, as an abundance of vaccination centers, and easy and free access to vaccination services in Saudi Arabia has changed many socioeconomic determinants of vaccination.

In the present study, the majority of the parents agreed that vaccination is safe and important to keep their children healthy and were strongly convinced that vaccination benefits exceed their harms. Several studies reported similar results from different countries [16,17,30]. Remarkably, our study showed a paradoxical finding, that a few of the respondents believed vaccination is prohibited in religion. Our findings superimposed views reported from a study conducted in Malaysia [17]. It is important to note that vaccination is consonant with the objectives of the Islamic religion and concepts, as declared by the Muslim religious scholars in the Islamic Organization for Medical Sciences. The pseudo-beliefs about the status of vaccines jeopardize the safety of children and expose communities as a whole [36].

Slightly more than 40% agreed that vaccination is associated with adverse effects. However, common local reactions like pain, redness, and swelling at the vaccine injection site and other systematic reactions, including fever, irritability, drowsiness, and rash may be associated with immunization [37]. Tagbo et al. showed that 20% of the mothers would not continue immunization if their child suffered any adverse effects [38]. An increased risk of narcolepsy was reported following vaccination with Pandemrix, a monovalent 2009 H1N1 influenza vaccine that was used in several European countries during the H1N1 influenza pandemic [39]. Thus, more attention should be focused on addressing narcolepsy as a severe vaccination adverse effect in future studies. Therefore, parents’ education about these side effects is imperative. This study revealed that a positive attitude is predicted by the female gender. This association between female gender and attitude towards child immunization could be justified as the mothers usually accompany their children during the vaccination visits, and may be keener to probe information from the internet or social media.

This study was conducted in a single city in Saudi Arabia; hence, the study findings cannot be generalized to Saudi parents in the entire country. Moreover, this study did not explore the vaccination status and compliance of the respondents. Furthermore, due to using convenience sampling, the majority of participants happened to have university level education.

## 9. Conclusions

In summary, our study findings revealed adequate knowledge and positive attitudes among our study sample to childhood vaccination. Continuous educational awareness campaigns are desirable to promote parents’ knowledge of immunization programs in the Kingdom.

## Figures and Tables

**Table 1:** Socio-demographic characteristics of Saudi parents (*N* = 1200).

Characteristics	*n* (%)
**Age (years)**	
18–29	167 (13.9)
30–39	481 (41.1)
40–49	310 (25.8)
≥50	242 (20.2)
**Gender**	
Male	615 (51.2)
Female	585 (48.8)
**Educational Level**	
Primary	29 (2.4)
Secondary	189 (15.8)
University	982 (81.8)
**Number of Children**	
1	283 (23.6)
2–3	499 (41.6)
>3	418 (34.8)

**Table 2:** Saudi parental knowledge about child immunization.

Knowledge Items	Yes	No	Don’t Know
Routine vaccination protects children from infectious diseases and their complications	1116 (93.0%)	47 (3.9%)	37 (3.1%)
First dose of vaccination is given at birth	1041 (86.8%)	73 (6.1%)	86 (7.2%)
Most diseases against which children are vaccinated occur during the first years of life	876 (73.0%)	177 (14.8%)	147 (12.3%)
Multi-doses of the same vaccine given at intervals are important for child immunity	838 (69.8%)	151 (12.6%)	211 (17.6%)
More than one vaccine at the same time has no negative impacts on child immunity	571 (47.6%)	296 (24.7%)	333 (27.8%)
Is it important to vaccinate children during immunization campaigns	923 (76.9%)	200 (16.7%)	77 (6.4%)
It is recommended to vaccinate children against seasonal influenza	674 (56.2%)	316 (26.3%)	210 (17.5%)
Immunization can cause autism	119 (9.9%)	715 (59.6%)	365 (30.4%)
Common colds, ear infection, and diarrhea are not contraindications for vaccination	388 (32.3%)	558 (46.5%)	254 (21.2%)

**Table 3:** Saudi parental attitudes on child immunization.

Attitudes Items	Strongly Agree + Agree *n* (*n*%)	Not Sure *n* (*n*%)	Strongly Disagree + Disagree *n* (*n*%)
Child immunization is important	1123 (93.7)	51 (4.3)	24 (2)
Immunization is more beneficial than harmful	1075 (89.7)	84 (7.0)	39 (3.3)
Vaccines for child immunization are safe	1002 (83.6)	159 (13.3)	37 (3.1)
Child immunization is prohibited in religion	96 (8.0)	140 (11.7)	962 (80.3)
Immunizations are associated with side effects	492 (41.1)	417 (34.8)	289 (24.1)
Child can become infected after immunization with the disease/s against which he/she was vaccinated	413 (24.5)	432 (36.1)	353 (29.5)
Compliance to immunization schedule is important	1110 (92.7)	54 (4.5)	34 (2.8)
Immunization keeps your child healthy	1090 (91.0)	75 (6.3)	34 (2.8)

**Table 4:** Association between demographic characteristics toward knowledge and attitudes on vaccination.

Characteristics	Knowledge on Vaccination			Attitudes on Vaccination		
	Good 883 (73.3)	Poor 315 (26.3)	*p*-Value	Negative 676 (56.4)	Positive 522 (43.6)	*p*-Value
**Age (years)**						
18–29 30–39 40–49 ≥50	136 (15.4) 372 (42.2) 213 (24.1) 161 (18.3)	30 (9.6) 106 (33.8) 97 (30.9) 81 (25.8)	<0.001	91 (13.5) 279 (41.3) 164 (24.3) 142 (21.0)	75 (14.4) 199 (38.3) 146 (28.1) 100 (19.2)	0.397
**Gender**						
Male Female	428 (48.5) 455 (51.5)	155 (49.2) 160 (50.5)	0.823	356 (52.7) 320 (47.3)	227 (43.5) 295 (56.5)	0.002
**Educational Level**						
Primary Secondary University	18 (2.0) 150 (17.0) 715 (81.0)	11 (3.5) 39 (12.4) 265 (84.1)	0.066	546 (80.8) 114 (16.9) 16 (2.4)	434 (83.1) 75 (14.4) 13 (2.5)	0.500
**Number of Children**						
1 2–3 >3	226 (25.6) 373 (42.2) 285(32.2)	57 (18.10) 126 (40.0) 133 (41.9)	0.002	157 (23.3) 279 (41.3) 239 (35.4)	126 (24.1) 219 (42.0) 177 (33.9)	0.855
